# Microwaved-Assisted Synthesis of Starch-Based Biopolymer Membranes for Novel Green Electrochemical Energy Storage Devices

**DOI:** 10.3390/ma16227111

**Published:** 2023-11-10

**Authors:** Paweł Jeżowski, Jakub Menzel, Hanna Maria Baranowska, Przemysław Łukasz Kowalczewski

**Affiliations:** 1 Institute of Chemistry and Technical Electrochemistry, Poznan University of Technology, 4 Berdychowo Str., 60-965 Poznań, Poland; jakub.menzel@put.poznan.pl; 2 Department of Physics and Biophysics, Poznań University of Life Sciences, 38/42 Wojska Polskiego Str., 60-637 Poznań, Poland; hanna.baranowska@up.poznan.pl; 3 Department of Food Technology of Plant Origin, Poznań University of Life Sciences, 31 Wojska Polskiego Str., 60-624 Poznań, Poland

**Keywords:** potato starch, biomembrane, energy storage, green chemistry, EDLC

## Abstract

The investigated starch biopolymer membrane was found to be a sustainable alternative to currently reported and used separators due to its properties, which were evaluated using physicochemical characterization. The molecular dynamics of the biomembrane were analyzed using low-field nuclear magnetic resonance (LF NMR) as well as Raman and infrared spectroscopy, which proved that the chemical composition of the obtained membrane did not degrade during microwave-assisted polymerization. Easily and cheaply prepared through microwave-assisted polymerization, the starch membrane was successfully used as a biodegradable membrane separating the positive and negative electrodes in electric double-layer capacitors (EDLCs). The obtained results for the electrochemical characterization via cyclic voltammetry (CV), galvanostatic charge with potential limitation (GCPL), and electrochemical impedance spectroscopy (EIS) show a capacitance of 30 F g^−1^ and a resistance of 2 Ohms; moreover, the longevity of the EDLC during electrochemical floating exceeded more than 200 h or a cyclic ability of 50,000 cycles. Furthermore, due to the flexibility of the membrane, it can be easily used in novel, flexible energy storage systems. This proves that this novel biomembrane can be a significant step toward ecologically friendly energy storage devices and could be considered a cheaper alternative to currently used materials, which cannot easily biodegrade over time in comparison to biopolymers.

## 1. Introduction

Biopolymers are currently one of the most pursued scientific topics. There is prevalent global pollution with plastics at the macro- and microscales; therefore, it is necessary to limit this kind of pollution in the environment. This is why, in recent years, so many studies have proposed that the use of biopolymers can compete with traditional plastic polymers in many crucial fields such as medicine [1,2,3], the food industry [4,5], safety [6], and energy storage [7,8,9].

The energy storage of biopolymers is especially interesting as their implementation can lead to novel green energy storage devices, for example, electrochemical capacitors with high power, moderate energy, and a very long lifetime that are still an interesting solution in terms of energy storage. Due to their variety of designs depending on the type of electrolyte and electrode material, biopolymers can find applications in most branches of industry from large-scale uninterrupted power supply stations, through medium-scale kinetic energy capture systems in the automotive industry, to small-scale applications in portable devices [10,11,12,13,14]. Currently, the majority of energy storage devices operate with electrolytes based on organic solvents, i.e., either lithium-ion batteries or hybrid devices [15,16,17,18,19]. Such a situation may lead to future problems concerning waste disposal due to the harmful character of the applied salts, solvents, or polymeric separators.

To counteract such a scenario, there are many research works devoted to the application of water-based electrolytes [20,21,22,23,24]. However, even with the application of environmentally friendly electrolytes, one cannot fully neglect the negative impact of the growing demand on new systems that lead to an increased production of electronic waste. While neutral aqueous electrolytes are considered non-harmful, there still remains the problem of separator and package recycling.

The application of gel electrolytes is widely studied both in the case of batteries and electrochemical capacitors [25,26,27,28,29,30,31]. In consequence of the application of solid-state and gel electrolytes, several advantages emerged, including an easier fabrication process; meanwhile, the separator and the electrolyte are of the same material, leading to improved safety, flexibility, and lowered gas generation. However, one also has to consider the deterioration in power output due to the limited ion transport properties of solid-state electrolytes.

The works on gel-based electrolytes can be differentiated into two stages: in situ polymerization with electrolyte and ex situ polymerization and impregnation of gel in an electrolyte. In situ polymerization is applied mostly in battery research, in which the electrolyte is prepared by the dissolution of lithium salts in the ion-coordination polymer [32,33,34]. Similarly, in the case of electrochemical capacitors, such a process is accomplished by the polymerization of poly(vinyl alcohol), PVA, and poly(ethylene oxide), PEO, in the presence of H_2_SO_4_, LiCl, or H_3_PO_4_. However, these electrolytes have low conductivity, and for their use in combination with PVA or PEO electrolytes, their corrosive character and toxicity can be limiting factors [28,35,36,37,38].

In the case of ex-situ-prepared gel electrolytes, one might consider the use of ionic-liquid-impregnated sodium alginate or chitosan membranes. These were the first biocompatible membranes prepared through polymerization in an aqueous solution, washing in alcohol, and drying, before impregnation with an ionic liquid [29,39,40].

In recent years, a new generation of biogel electrolytes has been widely studied, with the most popularly applied ones being gelatin, sodium alginate, chitin, chitosan, hydrocellulose, or agar [20,41,42,43,44,45,46]. In the case of electrochemical energy storage devices, the first biogel membranes were introduced by Ishikawa [25]. However, the increase in use of biocompatible electrolytes came with the application of in situ polymerization in aqueous electrolytes [47,48,49,50,51,52]. Beyond environmental friendliness, the advantage of this group of electrolytes is a high affinity toward carbon, resulting in a very good power performance. Due to easy preparation, accessibility, and suitability for application, the most frequently applied electrolytes are based on sodium alginate or agar. In both cases, the electrolyte is prepared by heating the gel precursor in the electrolyte solution and cast directly on the electrode surface for in situ polymerization.

To follow the progressing miniaturization of electronics, modern electrochemical capacitors need to be reliable, safe, and bend-resistant [53,54]. The application of liquid electrolytes does need to fulfill the safety requirements due to possible leakage and pressure increase during operation. In contrast, solid-state electrolytes do not require high-cost packaging materials or excessive sealing, which results in a comparatively smaller size, lower weight, and higher reliability. Moreover, the demand for flexible devices is now on the rise with a growing demand for flexible devices due to the popularization of bendable mobile phones, displays, and wearable electronics.

**Figure 1:**
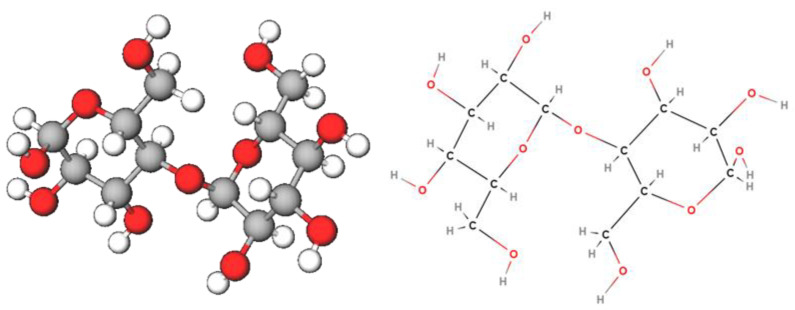
Representation of the starch molecule in 3D model (**left**) and full chemical structure (**right**).

In this work, a novel starch-based gel electrolyte for aqueous-based electrochemical capacitors is proposed. Starch, a naturally occurring carbohydrate that is abundant in various plant sources, finds extensive applications in different industries, which showcases its versatility and utility [55]. It is a natural polymer produced from amylose and amylopectin, two types of polysaccharides built of α-d-glucopyranosyl units connected by (1,4) and (1,6) bonds [56] (Figure 1). Due to its cellulosic features, starch is considered a promising low-environmental-impact material and is widely applied in the food, paper, and textile industries and many more [57,58,59,60,61]. Starch has also emerged as a pivotal player in cutting-edge technologies such as electric double-layer capacitors (EDLCs), commonly known as supercapacitors. In this domain, starch acts as a green binder [62] or a potential precursor material for activated carbon electrodes due to its high carbon content and porous structure [63,64]. The unique electrochemical properties of starch-derived activated carbon facilitate enhanced energy storage and quick charge-discharge cycles in EDLCs. As the push for eco-friendly and efficient energy storage solutions gains momentum, starch-derived electrodes offer a novel energy storage technology and promote a greener approach to electronic devices and energy systems.

Keeping the above in mind, the goal was to develop a cheap method of obtaining of the self-standing novel starch-based membrane to assess its mechanical properties and application in electrochemical capacitors. Starch biopolymers utilized in the energy storage demonstrate stable performance even after floating and cycling at a voltage of 1.6 V.

## 2. Materials and Methods

### 2.1. Starch-Based Membrane Preparation Procedure

**Figure 2:**
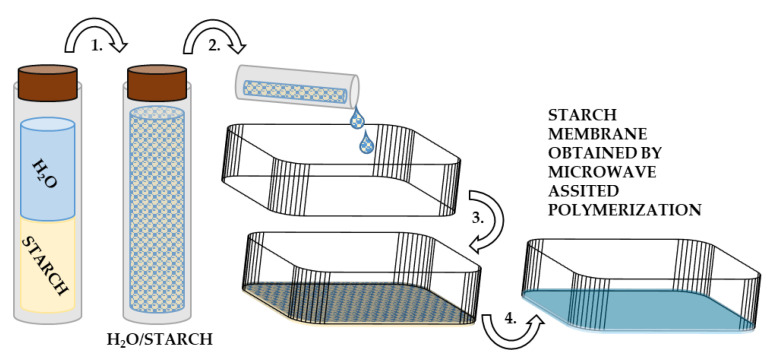
Schematic description of step-by-step preparation of biopolymer starch biopolymer: (1) creating of water and starch suspension, (2) transferring the suspension of water and starch into silicone box, (3) spreading a thin film of the suspension, (4) microwave-assisted polymerization.

Potato starch (Zetpezet Sp. z o.o., Piła, Poland) was placed in a glass vial containing distilled water in volumetric ratio of starch to water 1:1. It was shaken for 30 s at 2800 RPM so the suspension was uniform (VORTEX 1, IKA, Königswinter, Germany). The obtained suspension was placed at the bottom of a silicone box and spread to form a thin layer. The box with the suspension was heated in a microwave oven at power of 1000 W for 10 s. At the end, a semitransparent membrane was formed, which was then placed in an oven (UN30, Memmert GmbH + Co.KG, Schwabach, Germany) and preheated (to 80 °C, 1 h). After the membrane was dried, the measurement of thickness was carried out and it was estimated to be approximately 250 μm. Then, the biopolymer was immersed in a 1 M Li_2_SO_4_ solution and stored under room-temperature conditions until it was ready to be cut into separators using hollow punchers with a 12 mm diameter. The membranes did not dissolve in excess of the aqueous solution even after prolonged time of storage (12 months). An overview of the process for preparation of the starch biopolymer is depicted in Figure 2.

### 2.2. Assembling the Swagelok Cells

Electrodes intended for use in EDLCs were cut from the carbon cloth Kynol ACC-507-20 using hollow punchers with a 10 mm diameter. The electrodes had an average mass of roughly 8 mg ± 1 mg. Subsequently, these electrodes were placed in a Swagelok cell with current collectors made of stainless steel (316L), the surface of which was covered with conductive adhesive as described previously [62]. The electrodes were separated either by a starch separator (obtained as detailed in Section 2.1) or by a glass microfiber separator, 12 mm in diameter and 260 μm thick (Whatman^®^ GF/A, Sigma-Aldrich, Darmstadt, Germany), referred to as the GFA separator henceforth. Afterward, about 100 μL of electrolyte was added to each electrode and closed in the airtight body of the Swagelok cell. Prototype pouch cells were constructed to highlight the remarkable performance of the biopolymer membrane when subjected to external force during electrochemical testing, to showcase membrane flexibility.

### 2.3. Assays for Electrochemical Testing

To record all of the electrochemical data presented below, a VMP3 potentiostat/galvanostat (BioLogic, Seyssinet-Pariset, France) along with EC-Lab software version 11.32 (BioLogic, Seyssinet-Pariset, France) was used for data processing. For the qualitative description of the energy storage mechanism, cyclic voltammetry (CV) was used at various voltage ranges, from 1.0 to 2.0 V, at a sweeping rate of 5 mV s^−1^. This was carried out to determine the maximum operational voltage for subsequent electrochemical investigations, as determined by the S-value calculation method introduced by Weingarth et al. [65]. Subsequently, multiple scanning rates, ranging from 1.0 to 100 mV s^−1^, were employed at a voltage of 1.6 V. The quantitative assessment of the basic electrochemical parameters (capacitance, energy, and power) of the studied cells was performed via galvanostatic cycling with potential limitation (GCPL) conducted at 1.6 V, using a wide variety of currents from 100 to 5000 mA g^−1^. Additionally, the constant power (CP) technique was integrated into the investigations of electrochemical properties to accurately compute and draw a Ragone plot of the analyzed Swagelok cells [66]. The equivalent series resistance (ESR) and equivalent distributed resistance (EDR) were estimated via potentiostatic electrochemical impedance spectroscopy (PEIS) to assess the internal resistance (IR) of the tested Swagelok cells. These measurements were carried out across frequencies from 1 mHz to 100 kHz, using the input sinusoidal signal with amplitude of 5 mV. The resistance measurements during floating conditions were performed via the current interrupted (CI) value, which utilizes short current pulses separated by rest periods that enables the estimation of the internal resistance of the device. An accelerated aging assessment of the Swagelok cell was performed via the electrochemical floating test (EFT). This test involved maintaining the maximum voltage of the Swagelok cell for an estimated period of 2 h, which was repeated to evaluate its stability over the entire testing period. In parallel, the continuous cycling of the Swagelok cells was performed using the GCPL at 1.6 V and at current of 0.1 A g^−1^. Furthermore, self-discharge of the cell was characterized by first polarization of the cell to its maximal voltage and holding it for 2 h and later on leaving it in an open-circuit voltage for 24 h. All the recorded values of capacitance, energy, and power are presented per mass of both electrodes in the tested Swagelok cells. 

### 2.4. Raman and Infrared Spectroscopy

Raman spectra were recorded using the DXR3xi Raman Imaging Microscope (Thermo Fisher Scientific Inc., Waltham, MA, USA), which allows for the examination of both the structure and the texture of the samples. Photographic images were captured at two distinct magnifications, specifically 10× and 50×. The Raman spectra were recorded utilizing a laser with a wavelength of 532 nm and a power output in range from 1 to 5 mW. The spectral data were collected within a wavenumber range spanning from 400 to 3400 cm^−1^, and the spectra acquisition was performed at a magnification of 50×. The Nicolet iS5 FTIR Spectrometer (Thermo Fisher Scientific Inc., Waltham, MA, USA) was used to characterize the vibrational responses from the bonds in range of the wavenumber from 700 to 3600 cm^−1^.

### 2.5. LF NMR Relaxometry

First, a membrane sample with a known mass of approximately 1 g was weighed in glass weighing containers. Subsequently, the membrane underwent a hydration process using either distilled water (referred to as M1) or a 0.9% NaCl solution (referred to as M2). This hydration process occurred at a temperature of 20 °C until the samples gained stable mass for two consecutive days, which indicated complete hydration. Following this, all 0.25 g samples were placed in glass NMR tubes and sealed with Parafilm^®^. Measurements of the spin–lattice (T_1_) and spin–spin (T_2_) relaxation times were conducted using a pulsed NMR spectrometer (Ellab’ Poznań, Poland), which included an integrated temperature control system. The spectrometer operated at a frequency of 15 MHz, maintaining a temperature of 20.0 ± 0.5 °C. The T_1_ measurements used an inversion-recovery pulse sequence (π-TI-π/2), whereas the T_2_ relaxation times were determined using a pulse train of CPMG spin echoes (π/2-TE-(π)n). The repetition time between measurements was 10 s, and the number of spin echoes (n) was set at 100. 

## 3. Results and Discussion

**Figure 3:**
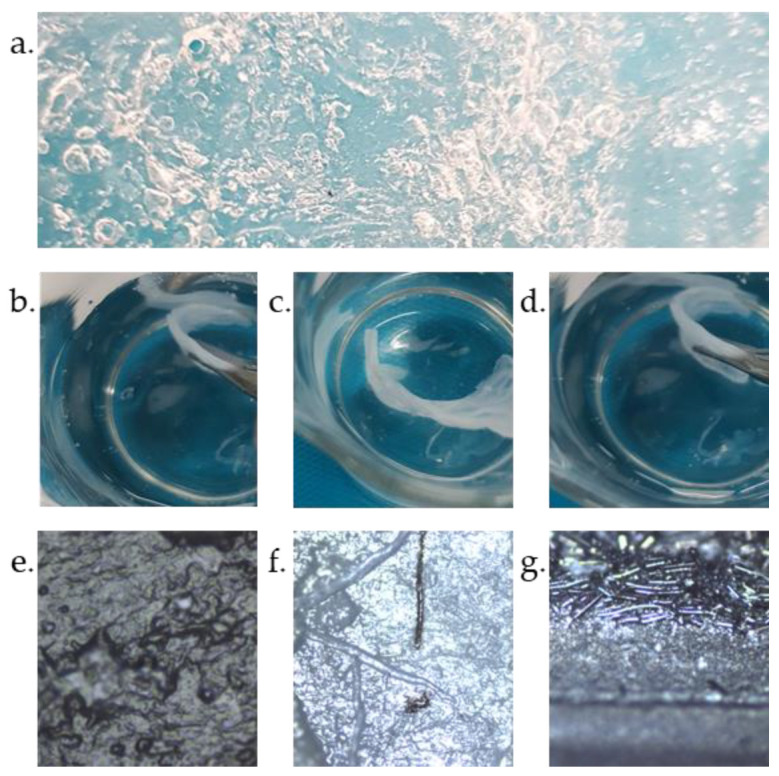
Photographic images of (**a**) dried membrane, (**b**–**d**) membrane soaked with water, and (**e**–**g**) microscopic images at magnification 50×.

The dried starch membrane (depicted in Figure 3a) was examined under a microscope to observe the changes that occurred following the microwave-assisted polymerization. The texture of the substrate differed substantially from that of the product, as it changed from the starch suspension to biopolymer membrane. This membrane, once prepared and hydrated, exhibited fascinating properties; it was no longer brittle and stiff, but extremely flexible, yet under an excess of external elongating stress it would break and could be easily cut and manipulated (Figure 3b–d). Additionally, it could be stored in electrolyte solution for a long time (ca. 12 months). However, if the membrane was wet and stored in moist conditions (ca. 80% humidity), it started to biodegrade only after a few days (Figure S1 in Supplementary File), which means it can be easily disposed of and should not have negative impacts on the environment.

Additionally, the preparation of the membrane is cheap, fast, easily reproducible, and scaled as one can prepare small samples of 4 cm^2^ as well as bigger ones (the largest piece prepared under laboratory conditions measured more than 100 cm^2^). Figure 3e–g display images of the membrane soaked with electrolyte and separating the carbon cloth electrodes. The texture of the biopolymer membrane became more uniform after wetting than the dry membrane. Furthermore, the membrane exhibited improved ability to adhere to the electrode surface and separated even the microscopic threads (Figure 3f) of carbon cloth, which furthermore improved the safety of an electrochemical device using such a membrane by limiting the possibility of short-circuiting. GFA separators are prone to cracking, tearing, or perforating under the same conditions.

**Figure 4:**
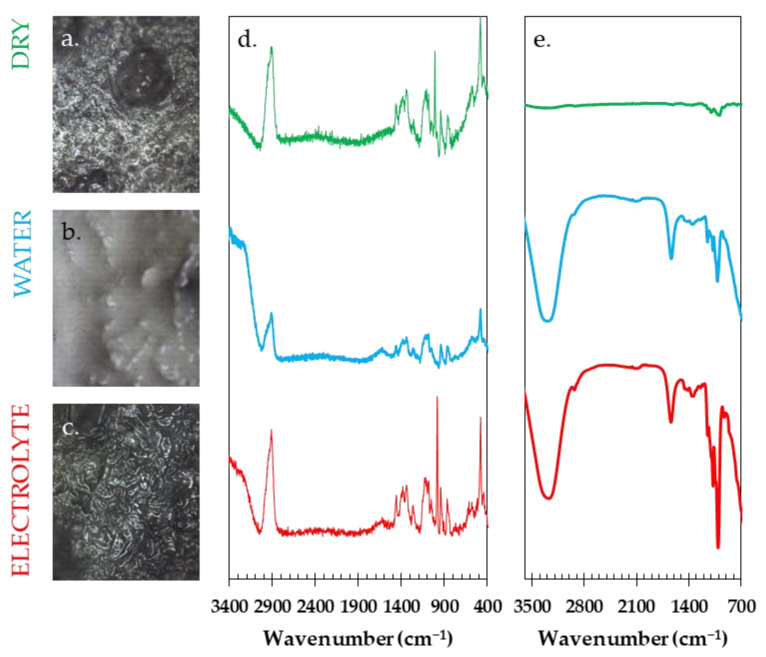
Microscopic images at 50× of (**a**) dried membrane and impregnated membrane with (**b**) water or (**c**) electrolyte together with their respective (**d**) Raman and (**e**) FTIR spectra. Colors of the Raman and FTIR spectra correspond to the dry membrane (green), membrane soaked in water (blue), or soaked with electrolyte (red).

The Raman spectra for the dry (green upper spectrum) and impregnated with water (blue middle spectrum) or electrolyte (red lower spectrum) starch membranes are shown in Figure 4d together with the representative microscope images from where the Raman spectra were taken (Figure 4a–c). The intensity changes above the wavelength of 3100 cm^−1^ can be largely attributed to varying amounts of solution present in the membrane. If the membranes were soaked overnight in water or electrolyte, their mass increased. After removing the excess of moisture with a paper towel, the mass of the soaked membrane increased almost three times in case of deionized water and almost two times for the electrolyte solution. The part of the spectrum near 2900 cm^−1^ can be assigned to the symmetric and antisymmetric -C-H stretching. The region near 1450 cm^−1^ usually corresponds to -C-H, -C-H_2_, and -C-OH deformations. In the range between 1350 and 1400 cm^−1^, we observe a coupling of the -C-CH and -C-OH deformation modes, whereas in the region between 1350 and 1100 cm^−1^, bands containing the contributions of several vibrational modes are observed. The band observed at 950 cm^−1^ can be assigned to the amylose linkage. A very strong signal near the 480 cm^−1^ confirms the presence of amylose and amylopectin in the structure of the chemical composition of the investigated material [67].

The complementary infrared studies (Figure 4e) of the dried starch membrane (green upper spectrum), membrane soaked with water (blue middle spectrum), or membrane soaked with electrolyte (red lower spectrum) show distinct features for the potato starch membrane; these include the broad and strong signal at ca. 3300 cm^−1^ that corresponded to the hydrogen bonds in the hydroxyl groups on the starch molecules, and symmetrical stretching vibrations of the -C-H_2_ band at 2900 cm^−1^. The visible bands near the values of wave number 1600 and 1400 cm^−1^ can be assigned to the asymmetric and symmetric vibration of -COO, whereas the signals within the range of 900 to 1200 cm^−1^ originate from the -C-O bands, along with additional bands corresponding to bending stretches [68]. Better visualization/magnification of the dry starch membrane FTIR spectrum can be found in the supplementary information (Figure S2). Both of the spectral characterization techniques prove that the obtained biopolymer membrane did not degrade or change its chemical structure during the microwave-assisted polymerization.

**Figure 5:**
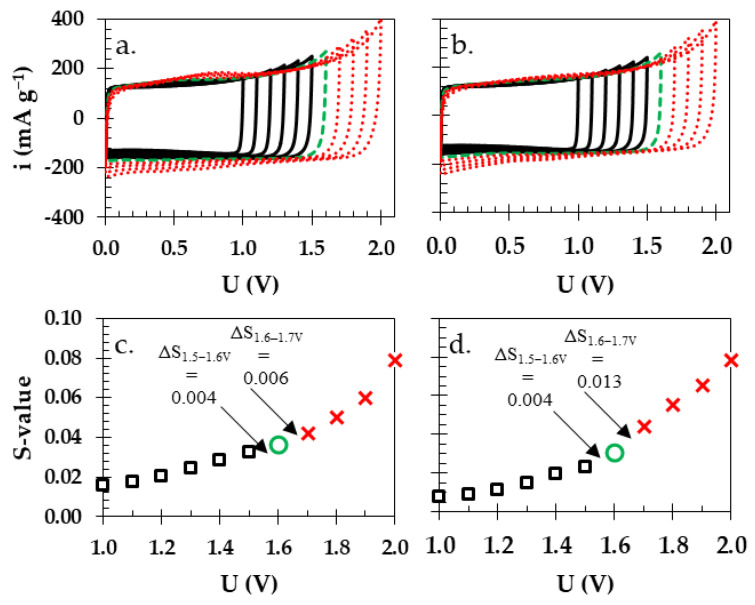
Cyclic voltammetry curves for electrochemical test of (**a**) starch biopolymer and (**b**) GFA separator. The color and type of the lines indicate stable voltage range (black solid curves), the maximum stable voltage (green dashed curve) exceeding the maximum stable voltage (red dotted lines). Estimated S-values for Swagelok cells with (**c**) starch biopolymer and (**d**) traditional GFA separator. The color and type of the markers indicate stable voltage range (black squares), the maximum stable voltage (green circles) exceeding the maximum stable voltage (red crosses).

Starch membrane soaked with an electrolyte solution (1 M Li_2_SO_4_) was placed between two carbon electrodes in a Swagelok cell. Figure 5a,b present the results of cyclic voltammetry studies conducted at a scan rate of 5 mV s^−1^ during incremental increases in voltage from 1.0 to 2.0 V. The cyclic voltammograms exhibit an almost perfectly rectangular shape characteristic of the predominantly capacitive mechanism of energy storage. Interestingly, when starch membrane was used, the rectangular shape of the cyclic voltammograms remained largely unchanged as the operational voltage increased until the value of 1.7 V, at which stage a peak at the positive polarization/anodic current (in range from ca. 0.6 to 1.0 V) and a peak at the negative polarization/cathodic current manifested. Similarly, a GFA separator displayed almost identical behavior and shape, where the increase in both anodic and cathodic currents was visible but less pronounced.

In order to accurately establish the maximum operational voltage for individual cells, the S-values were computed based on the CV data [65]. This involved integration of the curve surface area above (anodic) and below (cathodic) the zero value of the current (*y*-axis) at each of the applied voltage values. The S-value was then calculated as the ratio between two integral values (see Figure 5c,d). When the difference between the adjacent S-values (Δ) exceeds 0.005, that is the maximum voltage. On the one hand, the Swagelok cell with a GFA separator reached the maximum voltage of 1.6 V, because the ∆S-values between 1.6 V and 1.7 V exceeded the limit of the mentioned value (Δ = 0.013). On the other hand, for the Swagelok cell with the starch biopolymer, the ∆ S-values were 0.006 between the exact same voltages. This means that in both cases, the maximum voltage should be 1.6 V. In Figure 5a–d, the color and type of the lines indicate the stable voltage range (black solid curves, black squares), the maximum stable voltage 1.6 V (green dashed curve, green circles) for safely operating EDLC, and values exceeding the maximum stable voltage (red dotted lines, red crosses). The subsequent comparisons of both cells were performed at 1.6 V to continue our previous experiments [9,62].

**Figure 6:**
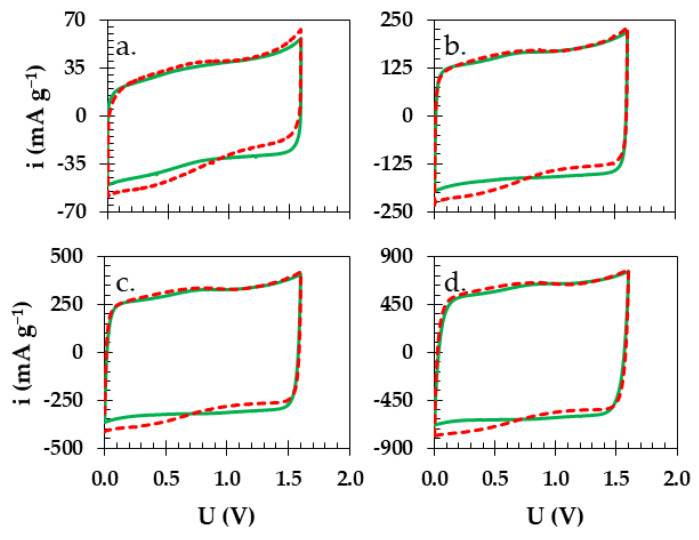
Comparison of cyclic voltammograms obtained during electrochemical testing of Swagelok cells with starch biopolymer (green solid line) and GFA separator (red dashed line) at (**a**) 1, (**b**) 5, (**c**) 10 and (**d**) 20 mV s^−1^, voltage 1.6 V.

The results from the CV analyses, a qualitative electrochemical technique, show that at the low sweeping rate of 1 mV s^−1^ (Figure 6a) for both Swagelok cells with different separators (green solid line—starch, red dashed line—GFA), the curves of similar shapes with the presence of an additional process were observed. This was particularly noticeable in case of Swagelok cell with GFA separator. The largely rectangular shape of the cyclic voltammograms of the cell with a starch biopolymer separator does not exhibit any major increase in current response. This confirms the observations from previous experiments, especially at the scanning rate of 5 mV s^−1^ (Figure 6b). As higher scanning rates were applied, the difference between the two devices remained pronounced. At 10 mV s^−1^ (Figure 6c), the rectangular shape for the biopolymer membrane persisted, and at 20 mV s^−1^ (Figure 6d), there was a comparably better charge propagation as well as stability when compared to the Swagelok cell with a GFA separator. 

**Figure 7:**
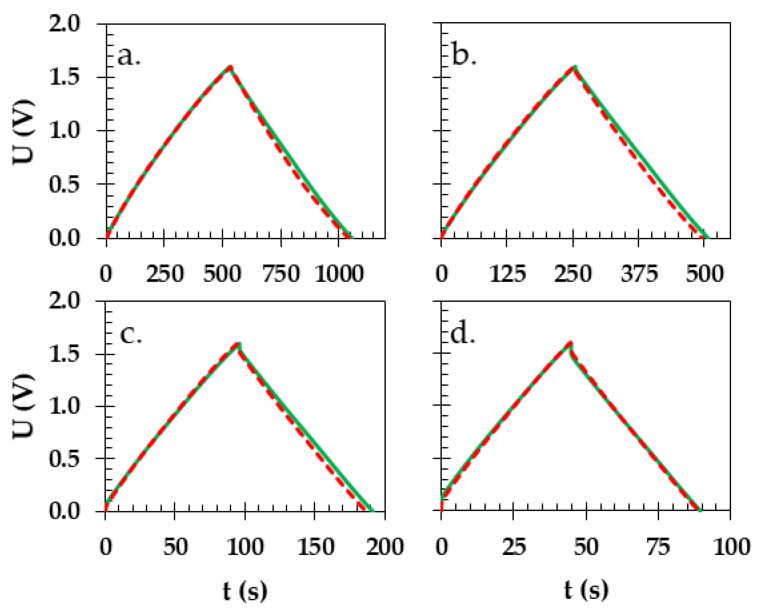
Charge/discharge profiles acquired from Swagelok cells with starch biopolymer (green solid line) and GFA separator (red dashed line) at specific currents of (**a**) 100, (**b**) 200, (**c**) 500, and (**d**) 1000 mA g^−1^.

The galvanostatic charge/discharge profiles provided additional quantitative information regarding the capacitive properties of the two Swagelok cells under investigation. Both of the cells had identical values of capacitance near 28 F g^−1^ in the presented range of the current densities using 100, 200, 500, and 1000 mA g^−1^ (Figure 7a–d, respectively). Remarkably, there was slight but visible deviation from the linear shape of charge/discharge for the GFA separator, especially under lower currents. The three-electrode investigations (Figure S3 in Supplementary Information) showed that under lower currents, the potential of the negative electrode for the GFA separators reached values of hydrogen evolution that might be not as detrimental at higher currents that are used in the case of EDLCs but could explain the observed differences in case of the two cells and the previous observations presented in this work.

**Figure 8:**
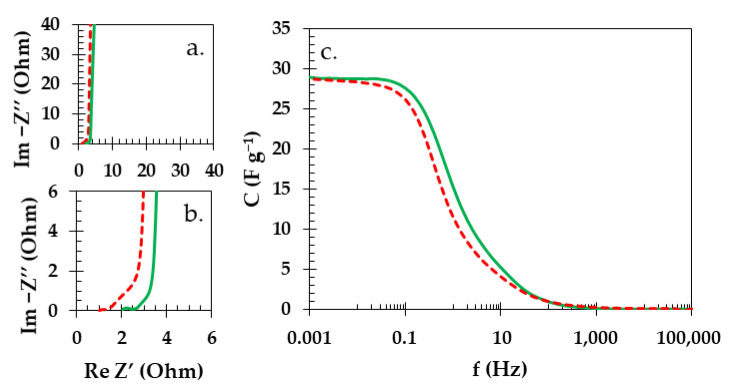
PEIS data represented as (**a**) Nyquist plot and enlargement (**b**) of the internal resistance region and (**c**) capacitance vs. frequency plot for Swagelok cell with starch biopolymer (green solid line) and GFA separator (red dashed line).

To give a more in-depth description of the charge propagation for the Swagelok cells with starch biopolymer or GFA separator, potentiostatic electrochemical impedance spectroscopy (PEIS) was used. As seen in the Nyquist plots (Figure 8a,b), both of the cells presented very similar characteristics. The equivalent series resistance (ESR) and equivalent distributed resistance (EDR) are the basic values that can be determined from this dataset. In the case of the ESR, the value was 2.0 Ω for the Swagelok cell with starch biopolymer and 1.0 Ω for the cell with GFA separator. The EDR values estimated from the plot were 2.7 Ω and 3.3 Ω for the Swagelok cell with starch biopolymer and GFA separator, respectively. While the difference between ESR values for the investigated cell was 1 Ω, the difference between EDR values was only 0.6 Ω, which was likely due the elongated part of the Warburg region (the part of the Nyquist plot that is tilted at the ca. 45 deg. Angle). The Warburg region is usually associated with mass transport limitations, and as in the case of an EDLC device, it is strongly related to the ion diffusion within the porous structure of an electrode material [69]. This was even more pronounced when the curve of the capacitance vs. frequency was plotted from the PEIS study (Figure 8c). Here, the capacitance value was comparably better retained by the Swagelok cell with biopolymer membrane. A smaller Warburg region, which represented noticeably shorter and better retention of the capacitance at higher frequencies in case of the Swagelok cell with starch biopolymer, can lead to an assumption that the use of a starch biopolymer that strongly adheres or impregnates or fuses with the electrode has beneficial effects on the electrochemical performance of the cell.

**Figure 9:**
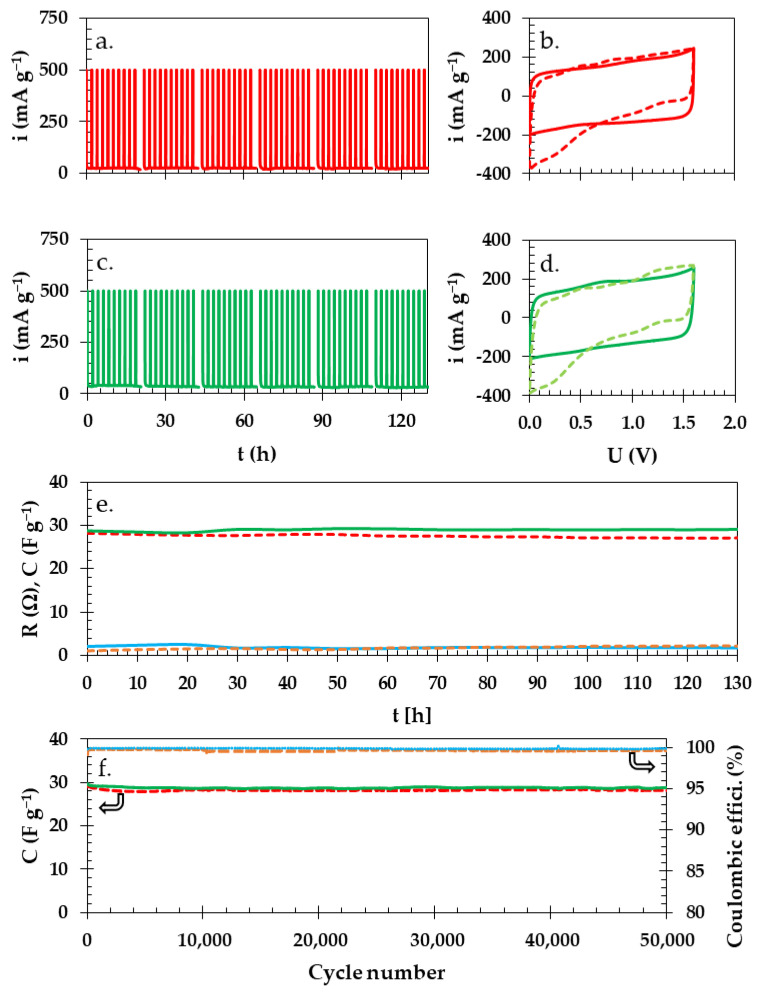
Curves of current leakage for Swagelok cells with (**a**) GFA and (**c**) starch biopolymer separator. CVs for (**b**) GFA and (**d**) starch separator before (solid line) and after (dashed lines) floating at 1.6 V. (**e**) Capacitance change (upper lines) and resistance (lower lines) during floating of Swagelok cells with starch biopolymer (solid lines) and GFA separator (dashed lines). (**f**) Continuous cycling for Swagelok cell with GFA separator (dashed lines) and starch biopolymer (solid lines) together with Coulombic efficiencies (upper lines) and capacitance values (lower lines).

To estimate the end-of-life criterion (drop of 20% in the value of capacitance in comparison to the initial value or two-fold increase in resistance), we measured the leakage current (Figure 9a,c) during which the maximum voltage was sustained for 2 h and then repeated consecutively for five times. After this, d additional electrochemical characteristic such as CV (Figure 9b,d), current interrupted (CI), and GCPL were performed during another 2 h to estimate the changes in qualitative energy storage and quantitate resistance and capacitance, in that order (Figure 9e). All of the mentioned measurements took approximately 22 h (which constituted a full cycle). Figure 9a,c present the total time of holding at maximum voltage for 120 h.

A leakage current experiment showed that the current necessary to uphold the voltage of 1.6 V was ca. 30 mA g^−1^ for the Swagelok cell with starch biopolymer, whereas in the case of the cell with the GFA separator, it was 23 mA g^−1^, as their respective dielectric properties are different. The initial 120 h of experiments did not reach the failure criterion (20% drop in capacitance) for either of the investigated Swagelok cells. Accordingly, the time of the experiment was prolonged by the repetition of the described schedule until after ca. 380 h at 1.6 V, both of the Swagelok cells, including the one with the starch biopolymer and the one with the GFA separator, reached their end-of-life criteria. After 200 h of floating, the CVs (dashed line) changed their shape noticeably in comparison to their initial state (solid line). This indicated deterioration of the used components in the Swagelok cells (Figure 9b,d), which could be caused by the partial oxidation and reduction processes taking place at the electrodes; such observations have been reported elsewhere [70].

During the floating experiment, the capacitance of the Swagelok cell with thestarch biopolymer barely changed and was approximately 29 F g^−1^. Similarly, the resistance remained at 2 Ω and in comparison, the capacitance of the Swagelok cell with the GFA separator showed a noticeable drop from 29 to 28 F g^−1^ (ca. 4%). At the same time, the overall resistance of the Swagelok cell with the GFA separator rose from 1.0 to 1.2 Ω (20%). The overall drop in capacitance and the increase in resistance in the case of the Swagelok cell with the GFA separator are in favor of a starch biopolymer as a separator, as it has more stable performance.

The data from the cycling of the Swagelok cells at the electric current regime of 0.1 A g^−1^ at 1.6 V show that there were almost no changes in capacitance during 50,000 cycles (Figure 9f). This proves that both the analyzed Swagelok cells with the starch biopolymer (solid lines) or with the GFA separator (dashed lines) had very stable and similar electrochemical characteristics. On the one hand, the Swagelok cell with GFA separator (dashed lower red line) showed a noticeable 7% drop in capacitance value after 50,000 cycles despite a Coulombic efficiency of 99.6% (dashed orange upper line). On the other hand, the Swagelok cell with starch biopolymer separator had an only 3% drop in capacitance (29 F g^−1^), whereas the Coulombic efficiency was ca. 99.8%.

**Figure 10:**
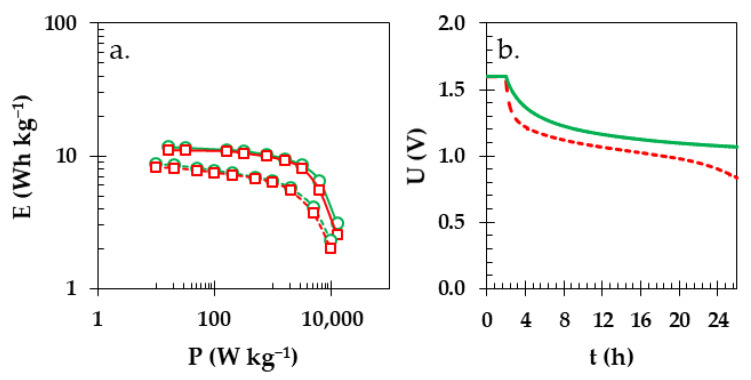
(**a**) Ragone plot energy vs. power calculated from GCPL (solid lines) and CP (dotted lines) for Swagelok cells with GFA (red line with square markers) and starch biopolymer separator (green line with circle markers). (**b**) Self-discharge of Swagelok cell with GFA (red dashed line) and starch biopolymer (green solid line) separator.

Both analyzed Swagelok cells presented virtually identical values of energy and power on a Ragone plot (Figure 10a) for the GFA separator (red lines with square markers) or starch biopolymer (green lines with circle markers). The respective values were calculated from the galvanostatic charge/discharge profiles (solid lines) or were based on the constant power technique (CP; dashed lines) which allows us to avoid overestimations of the calculated values and is more representative for real-life applications. A specific energy of ca. 8 Wh kg^−1^ could be maintained up to 1 kW kg^−1^ of specific power in case of both the starch biopolymer and the GFA separator. The noticeable drop in specific energy starts above 2 kW kg^−1^ of specific power. Interestingly, the implementation of a starch biopolymer in the construction of EDLC noticeably decreased the self-discharge of the Swagelok cells (Figure 10b). For the GFA separator (red dashed line), the drop in voltage during the 24 h of the OCV period was close to 48% (decrease from value of 1.60 to 0.83 V), whereas for the starch biopolymer it was 33% (decrease from value of 1.60 to 1.07 V). This means that the use of starch biopolymer substantially improved the usefulness of the EDLC energy storage device as well as the limits on the main drawbacks of EDLCs.

**Figure 11:**
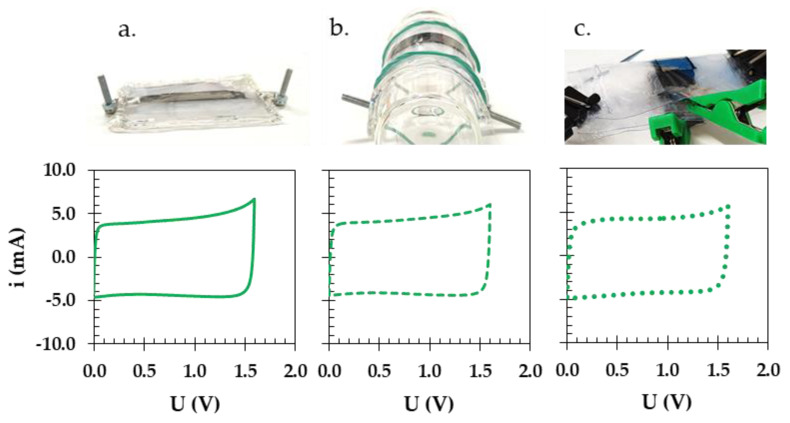
Pictures of the EDLC pouch cell prototype and corresponding CVs of starch biopolymer at various degrees of external stress (**a**) in neutral (solid line), (**b**) bent (dashed line), and (**c**) folded (dotted line) positions.

Finally, the flexibility of the starch biopolymer in the laboratory prototype of the pouch cell was investigated by applying external stress to the body of the prepared electrochemical device. As seen in Figure 11a–c, during the bending and folding, the electrochemical characteristics did not change significantly from the neutral position (solid CV curve) to the bent position (dashed CV curve). Only when the device was folded (dotted CV curve) did the charge propagation of the device decrease.

Due to the use of the obtained biomembrane in aqueous systems, it seems advisable to characterize the behavior of the membrane after its hydration. LF NMR is a non-invasive and non-destructive method used to analyses the molecular water behavior in various biological samples [71,72,73,74]. This technique entails the assessment of the spin–lattice T_1_ and spin–spin T_2_ relaxation times, which depict the motions of water molecules within the examined sample. Alterations in these values indicate shifts in the overall water dynamics concerning both unbound water (T_1_) and bound water, offering insights into the molecular behavior of these distinct water components [75]. Changes in molecular dynamics are also particularly important due to starch the retrogradation process [76]. An analysis using the LF NMR method allowed us to assess the stability of systems containing starch and thus assess the properties of the membrane obtained from it. The membrane was analyzed after hydration using distilled water (M1) and 0.9% NaCl solution (M2). The use of NaCl solution caused the hydrated membrane to gain a comparably higher weight after complete hydration vs. the membrane hydrated in distilled water (6016% vs. 3677%, respectively).

**Figure 12:**
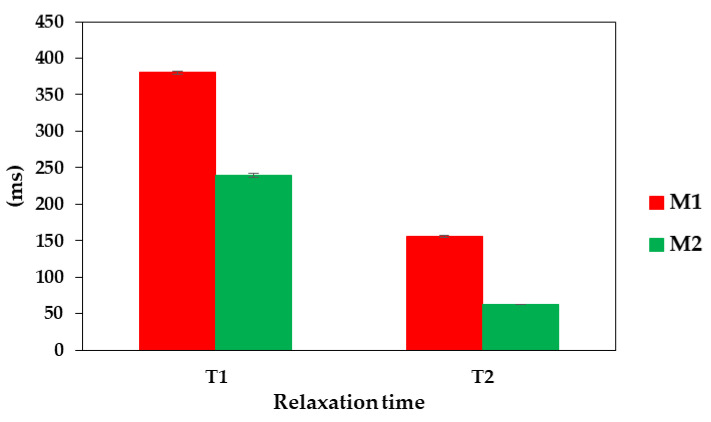
Results of LF NMR relaxometry measurements of the membrane hydrated in water (M1) and 0.9% NaCl solution (M2).

The observed longer values of spin–lattice times for M1 may be the result of relatively worse interactions of pure water with the biomembrane structure (Figure 12). As a result of the weaker interactions between water and the membrane, water created a relatively worse environment for the membrane and, as a result, its durability may have decreased. Also, the values of spin–spin relaxation times for M1 were significantly (almost four times) larger than for M2. This confirmed the conclusion that storage in water results in the formation of a less compact membrane structure after the hydration process. The T_2_ time results obtained for M2 show that it was a less relaxed system and therefore much more stable from the molecular standpoint. The use of such an obtained membrane in systems containing electrolyte solutions will result in the accretion of a stable biomembrane that can be used in energy storage devices such as EDLCs.

## 4. Conclusions

It is possible to prepare a fully ecological friendly membrane via the microwave-assisted polymerization of starch. This process itself did not utilize any complex techniques, expensive aperture, or costly chemicals or substrates in contrast to some of the other reports present in the literature. One of the first applications proposed and investigated here was the separation of the electrodes in energy storage devices such as EDLCs. With a capacitance of 30 F g^−1^, resistance of 2 Ω, and longevity of 50,000 cycles, this implementation was successful as the starch biopolymer showed its electrochemical performance almost identically to the usually used GFA separators. In some aspects, including mass transfer of ions or self-discharge, a noticeable improvement in comparison to the traditional separators was noted. Overall, this starch biopolymer can be a feasible alternative for next-generation green energy storage devices with even slightly better electrochemical performance than the currently used GFA separators. Moreover, the superior flexibility of the starch biopolymer could be a step towards the flexible energy storage devices for wearable electronics as the electrochemical characteristics in the self-made electrochemical pouch cell did not change upon applied external stress to the Swagelok cell.

## Data Availability

The datasets generated for this study are available on request to the corresponding author.

## Supplementary Materials

The following supporting information can be downloaded at: https://www.mdpi.com/article/10.3390/ma16227111/s1, Figure S1. Photographic images of the starch biopolymer before and after 7 days; Figure S2. Photographic images of the starch biopolymer before and after 7 days; Figure S3. Three electrode galvanostatic charge/discharge profiles for the electrochemical with a) starch biopolymer and b) GFA separator at 0.05 A g^−1^ with maximum voltage of 1.6 V.
