# Supplementary material for: Microwaved-Assisted Synthesis of Starch-Based Biopolymer Membranes for Novel Green Electrochemical Energy Storage Devices
Source: Materials (Basel). 2023 Nov 10;16(22):7111. doi: 10.3390/ma16227111 (PMC10672333; doi:10.3390/ma16227111)
Supplement: Supplementary file 1 [file materials-16-07111-s001.zip › materials-2668927-supplementary.pdf]

materials

Supplementary Information

Microwaved-Assisted Synthesis of Starch-Based Biopolymer Membranes for Novel Green Electrochemical Energy Storage Devices

Paweł Jeżowski [1,*], Jakub Menzel [1], Hanna Maria Baranowska [2] and Przemysław Łukasz Kowalczewski [3,*]

[1] Institute of Chemistry and Technical Electrochemistry, Poznan University of Technology, 4 Berdychowo Str., 60-965 Poznań, Poland; jakub.menzel@put.poznan.pl, pawel.jezowski@put.poznan.pl
[2] Department of Physics and Biophysics, Poznań University of Life Sciences, 38/42 Wojska Polskiego Str., Poznań, 60-637, Poland; hanna.baranowska@up.poznan.pl
[3] Department of Food Technology of Plant Origin, Poznań University of Life Sciences, 31 Wojska Polskiego Str., 60-624 Poznań, Poland; przemyslaw.kowalczewski@up.poznan.pl
* Correspondence: pawel.jezowski@put.poznan.pl, przemyslaw.kowalczewski@up.poznan.pl

Biodegradation of the Starch Membrane

The starch membrane was left for the period of time of seven days in the box with humidity of ca. 90% after that time membrane was removed and photographed to confirm natural biodegradation of the material which was visible by the formation of brown spots on the material (Figure S1).

a. b.

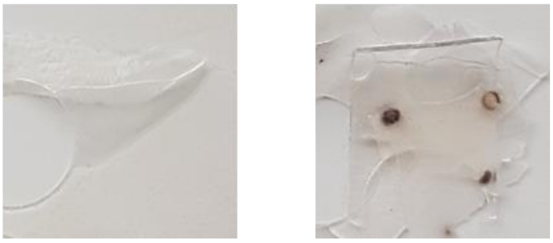

Figure S1. Photographic images of the starch membrane before and after 7 days.

Magnification of FTIR Spectrum for Dry Starch Membrane

For better visibility of all bands the FTIR spectrum is presented in Figure S2.

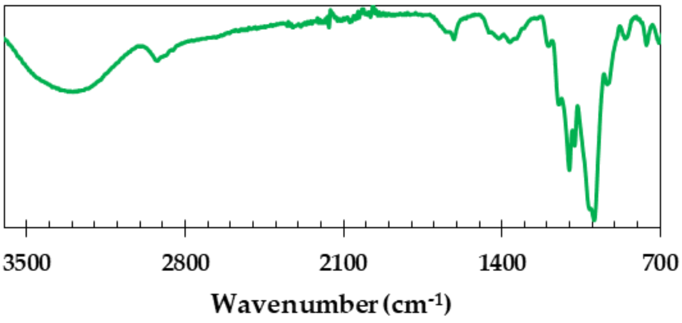

Figure S2. Photographic images of the starch membrane before and after 7 days.

Three Electrode Investigation of Electrochemical Cells

Reference electrode mercury/mercurous sulfate (Hg/Hg_2SO_4) reference electrode filled with 0.5 mol L^{-1} H_2SO_4 (potential 0.674 V vs. NHE) was introduce to the laboratory cell for the observation of potential change of individual electrodes: positive electrode (red dotted line), negative electrode (blue dashed line) as well as overall voltage of the electrochemical cell (black solid line) as it is seen in the Figure S3a the potential profiles of positive electrode and negative electrode in case of the electrochemical cell with starch membrane are within stability limits of oxygen and hydrogen evolution (+0.8 and −0.8 V vs. ref. NHE). While in the case of electrochemical cell with the glass fiber separator the negative electrode reaches the hydrogen evolution limit for the negative electrode. This data could indicate while in the case of the cells with glass fiber separator the electrochemical performance deteriorates with the time even if there is layer of conductive glue securing the current collectors from the corrosion.

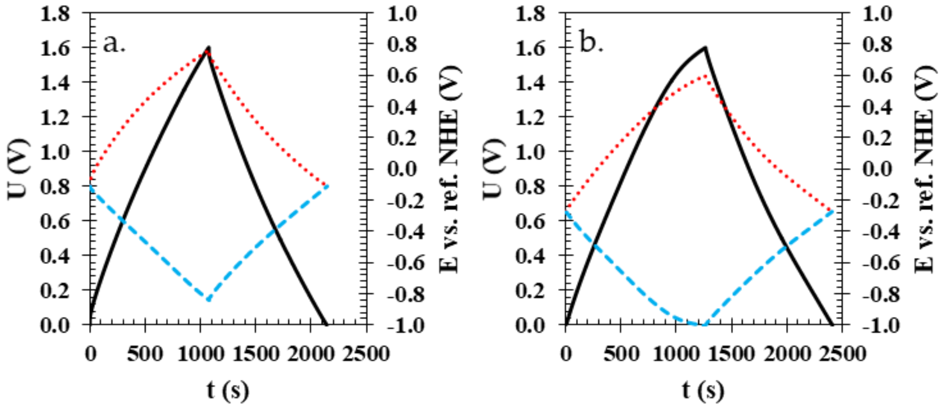

Figure S3. Three electrode galvanostatic charge/discharge profiles for the electrochemical with (**a**) starch membrane and (**b**) glass fiber separator at 0.05 A g^{-1} with maximum voltage of 1.6 V.
